# A Critical Case of Chronic Eosinophilic Leukemia: Diagnostic Criteria and Response to Cytarabine

**DOI:** 10.1155/crom/8887007

**Published:** 2025-09-27

**Authors:** Ana Karen Cruz-Acevedo, Eduardo L. Pérez-Campos, María Teresa Hernández-Huerta, Miguel Cruz-Reyes, Joel López-Matías, Laura Pérez-Campos Mayoral, Víctor Cruz-Hernández

**Affiliations:** ^1^Research Centre-Faculty of Medicine, National Autonomous University of Mexico-Benito Juárez Autonomous University of Oaxaca, Oaxaca, Mexico; ^2^National Technological of Mexico, Technological Institute of Oaxaca, Oaxaca, Mexico; ^3^Secretariat of Science, Humanities, Technology and Innovation SECIHTI, Faculty of Medicine, Benito Juárez Autonomous University of Oaxaca, Oaxaca, Mexico; ^4^Internal Medicine Department, General Hospital Dr. “Aurelio Valdivieso” IMSS-Bienestar, Oaxaca, Mexico; ^5^Research Department of General Hospital Dr. “Aurelio Valdivieso” IMSS-Bienestar, Oaxaca, Mexico

**Keywords:** cytarabine, cytarabine response, cytogenetic alterations, myeloproliferative neoplasms, organ damage, ulcerative lesions

## Abstract

Chronic eosinophilic leukemia (CEL) belongs to the group of chronic myeloproliferative neoplasms characterized by the persistence of an absolute eosinophil count (AEC) > 1.5 × 10^9^/L for 1–6 months and is accompanied by organ damage. The new World Health Organization criteria for the diagnosis of CEL are the presence of the cytogenetic alteration *FIP1L1::PDGFRA* as an oncogene; in its absence, morphological criteria in bone marrow define the diagnosis with blasts > 5% and < 20% and in peripheral blood with > 2% of blasts and eosinophilia >1.5 × 10^9^/L. The current study describes the case of a 60-year-old man who was admitted to the internal medicine department in critical condition with fever, cough, dyspnea, shortness of breath, and intense abdominal pain, and his spleen had the following measurements: 8-8-10 cm. The complete blood count showed hemoglobin 8.5 g/dL, platelets92 × 10^9^/L, leukocytes105.97 × 10^9^/L, total neutrophils31.79 × 10^9^/L, AEC69.40 × 10^9^/L, and lymphocytes4.23 × 10^9^/L, and the bone marrow analysis revealed 25% eosinophils and 12% myeloblasts. Thorax and abdomen computed tomography showed interstitial infiltrate, pleural effusion, and splenomegaly. Endoscopy showed ulcerative lesions in the digestive tract. This case underscores the crucial role of both bone marrow and peripheral blood morphological criteria in diagnosing CEL. This rare disease manifests at an advanced stage with complex clinical features but responds well to cytarabine.

## 1. Introduction

Eosinophils differentiate from hematopoietic progenitor cells through interleukin-3 (IL-3) and interleukin-5 (IL-5) and granulocyte-macrophage colony-stimulating factor (GM-CSF). The normal eosinophil count ranges from 0.10 to 0.50 × 10^9^/L. A slight increase is defined as 0.50–1.50 × 10^9^/L, a moderate increase is defined as 1.5–5.0 × 10^9^/L, and a severe increase is more than 5.0 × 10^9^/L. These cells belong to the immune system and respond to parasitic infections, cancer, and allergies such as asthma, rhinitis, and dermatological atopies. Hypereosinophilia (HE) is characterized by an increase in eosinophils >1.5 × 10^9^/L and may be present in any of the above conditions. Hypereosinophilic syndrome (HES) is characterized by the persistence of an absolute eosinophil count (AEC) > 1.5 × 10^9^/L in two or more determinations for 1–6 months [1]. The World Health Organization (WHO) classifies HES as primary, reactive/secondary, and idiopathic. Primary HES corresponds to chronic myeloproliferative neoplasia and is associated with lymphoid neoplasia. Myeloproliferative HES (M-HES) is classified into chronic eosinophilic leukemia (CEL) and HES myeloid/lymphoid; approximately 20%–25% of them present some oncogenes [2]. Here, we report a case of CEL with a negative gene, which fulfills this rare pathology's clinical and morphological criteria.

## 2. Case Report

**Figure 1:**
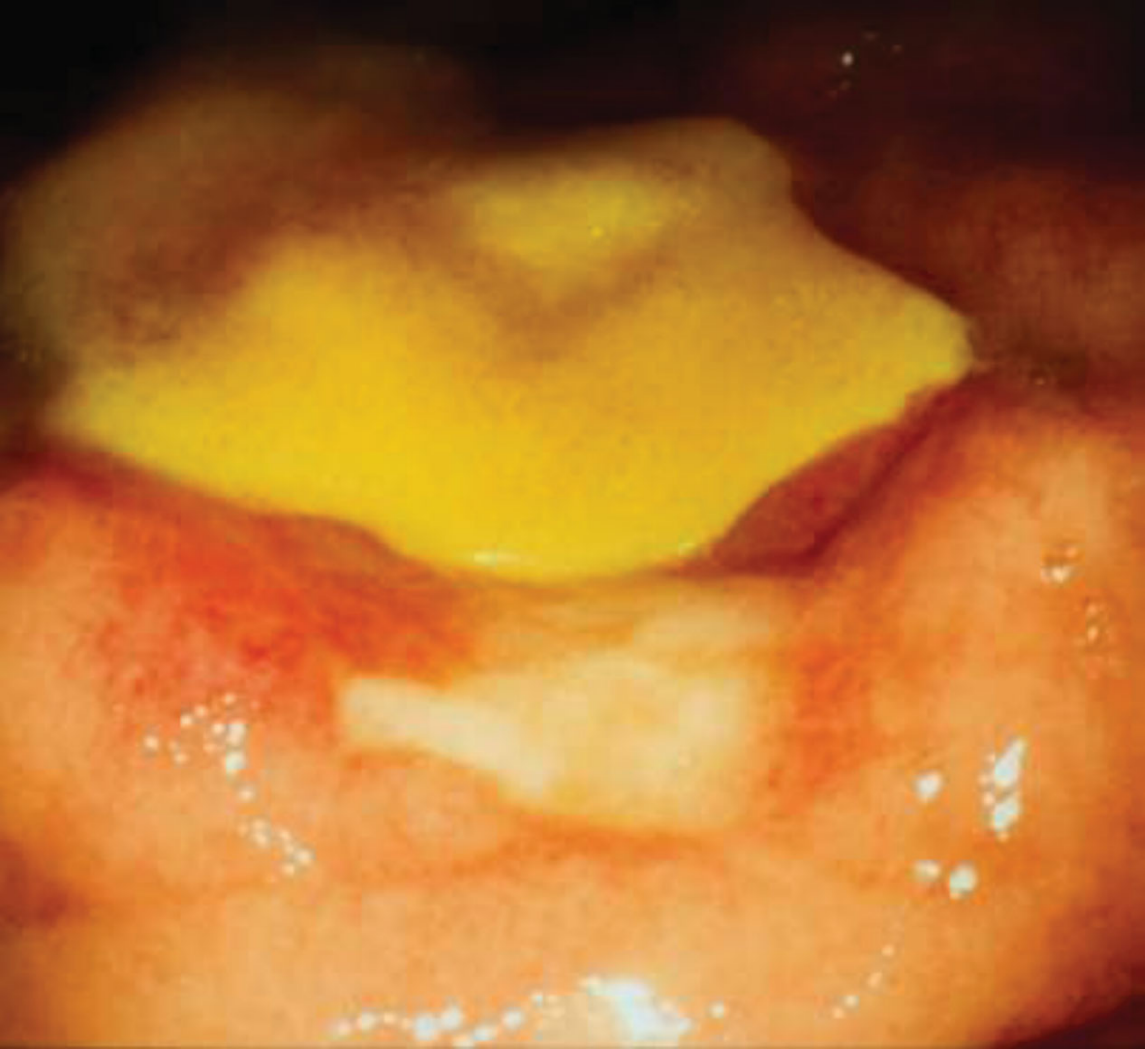
Ulcerative lesions in the intestinal mucosa.

A 60-year-old male, originally from La Sierra, Oaxaca, Mexico, who works as a carpenter and is therefore exposed to solvents such as thinner, received medical attention in August 2017. He had arthralgia for the last 3 months and was treated with methotrexate. He also had inflammatory bowel disease, and a panendoscopy revealed ulcerations in the stomach, duodenum, colon, and anus, as well as proctitis (Figure 1).

The patient reports severe abdominal pain rated 8/10 on the visual analogue scale, accompanied by constitutional symptoms of marked fatigability and pallor. The review was also positive for dyspnea with a productive cough, diffuse arthralgias, and generalized dryness of the skin and mucous membranes. Notably, despite the severe pain, he denied any associated abdominal rigidity, distension, nausea, or vomiting. The remainder of the comprehensive systemic inquiry was negative, review of the cardiovascular, genitourinary, and neurological systems was unremarkable, and he specifically denied any chest pain, palpitations, urinary symptoms, bleeding or bruising, lymphadenopathy, or deficits in vision, hearing, taste, or smell.

**Figure 2:**
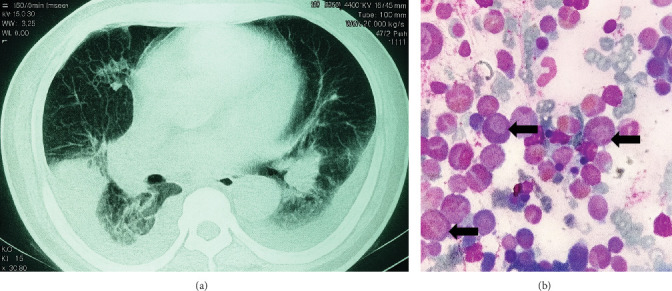
(a) Thoracic computed tomography showing the lungs with diffuse interstitial infiltrates and pleural effusion. (b) Bone marrow aspirate demonstrating myeloid hyperplasia (black arrows) and hypereosinophilia.

Histopathology report: Mucous infiltrated by neutrophils and eosinophils was observed, and probable chronic ulcerative colitis was treated with mesalazine for 1 week without response. The patient was referred in critical condition and hospitalized with fever, cough, dyspnea, yellow phlegm, severe respiratory difficulty, and intense abdominal pain. Physical exploration: Temperature is 38.5°C (311.65°K), blood pressure is 100/60 mmHg (13.3/8.0 kPa), respiratory rate is 38 breaths/min, heart rate is 90 beats/min, and oxygen saturation is 90% (fractional concentration 0.90). Lung fields: Both presented with crackles. Abdomen: intense pain, increased peristalsis, splenic measurements 8-8-10 cm, and a nonpalpable liver. Blood tests were required and are presented in Table 1. The echocardiogram is normal. Thoracoabdominal computed tomography shows pneumonia and pleural effusion (Figure 2); and the bone marrow (BM) aspirate reveals myeloid hyperplasia, with 12% myeloblasts and 25% eosinophils (Figure 2).

The first diagnosis was chronic myeloid leukemia (CML) in the accelerated phase. The reverse transcriptase–polymerase chain reaction (RT-PCR) of *BCR::ABL1* fusion resulting from t(9;22)(q34;q11) and *FIP1L1::PDGFRA* fusion from chromosome 4q12 was negative. The patient required mechanical ventilation for 7 days, antibiotic coverage with amikacin/vancomycin, and transfusion of two units of red blood cells (RBCs). CML was discarded, and the patient was treated as CEL. For treatment, the patient received cytarabine 100 mg/m^2^/day for 4 days. On the 7th day, the patient showed improvement, allowing removal of mechanical ventilation, with a decrease in abdominal pain and spleen size. In addition, control studies were requested: hemoglobin 10.5 g/dL, leukocytes 19.86 × 10^9^/L, absolute neutrophils 8.31 × 10^9^/L, AEC 9.81 × 10^9^/L, and platelets 120 × 10^9^/L. After 3 weeks, the patient was discharged due to an improvement in the AEC to 1 × 10^9^ L^−1^. Maintenance treatment was hydroxyurea 1 g oral administration every 24 h, and a 24-month follow-up was scheduled, monitoring a mean AEC between 1 and 2.5 × 10^9^/L. Nonetheless, the patient abandoned his medical appointments, and attempts by social services to contact him were unsuccessful.

## 3. Discussion

Both CEL and HES myeloid/lymphoid belong to M-HES but are differentiated because each expresses a different gene. CEL is defined by sustained HE (eosinophil count ≥1.5 × 10^9^/L and ≥ 10% eosinophils) for 4 weeks, the presence of a clonal abnormality, abnormal BM morphology, or the presence of increased blasts (≥ 5% in the BM and/or ≥ 2% in the peripheral blood) [3]. Additionally, there is the presence of the *FIP1L1::PDGFRA* fusion resulting from the deletion of Chromosome 4 (q12q12) [4–7]. However, these studies are difficult to access, so the diagnosis tends to be defined by morphological criteria in peripheral blood with cy > 2% of blasts and BM with myeloblasts > 5% but < 20%. For the epidemiological aspect of CEL, Wang et al. [8] analyzed a database of 487 patients from 2001 to 2022 in the United States; they observed an annual incidence of 0.03 cases per 100,000 inhabitants, an average age of 57 years, and a male predominance; regarding ethnicity, the majority were White (73.9%), with the remainder being African American, Asian, and Native American [9].

The *FIP1L1::PDGFRA* gene gives rise to a constitutively active fusion tyrosine kinase by joining the N-terminal domain of FIP1L1 with the C-terminal kinase domain of *PDGFRA* [10]. The *FIP1L1::PDGFRA* gene is responsible for the uncontrolled proliferation of eosinophils and suppression of apoptosis; at the same time, the gene is sensitive to the tyrosine kinase inhibitor (TKI) imatinib [9]. Eosinophils hold several chemical mediators such as the primary basic protein, eosinophil-derived neurotoxin or Ribonuclease 2, eosinophil cationic protein or Ribonuclease 3, and eosinophil peroxidase [11]. All these chemical mediators have immunological and inflammatory activity under physiological conditions; nonetheless, in their neoplasm presentation, they can cause severe tissue damage and even death.

The clinical manifestations of CEL are due to the infiltration of eosinophils and massive release of chemical mediators into the tissues (Loeffler syndrome), affecting the heart (endocarditis), lungs (pneumonia), and digestive tract with gastritis, enteritis, diarrhea, and ulcers. Cutaneous manifestations are rare but may include erythema, pruritic papules, and nodules. Other affected organs include the brain, liver, and bile ducts [8, 12]. In the presented case, the lungs, digestive tract, spleen, and joints were the organs most affected. Cytarabine was used during the critical phase and had a good hematological response with a decrease in AEC, so the symptoms and the size of the spleen were reduced.

Cytarabine, also known as cytosine arabinoside (1-*β*-d-arabinofuranosylcytosine), is a deoxycytidine nucleoside analogue. It is recognized as one of the most effective antineoplastic agents in both initial and salvage treatments for myeloid and lymphoid leukemias, as well as for Hodgkin and non-Hodgkin lymphomas [13]. Labeled uses of cytarabine include the treatment of acute lymphoblastic leukemia, remission induction therapy for acute myeloid leukemia in both adult and pediatric patients, management of CML in the blast phase, and prophylaxis and treatment for meningeal leukemia [14].

The main differential diagnosis of CEL is CML, which can also present with HE but can be differentiated by the presence of the Philadelphia chromosome, t(9;22) (*BCR::ABL* clone), which is found in 95% of cases. Another differential diagnosis is idiopathic HES, which is characterized by the absence of a clone. However, the WHO and experts include morphological criteria such as the presence of blasts < 2% in peripheral blood and < 5% in BM [9, 12, 15].

When CEL presents the positive gene (*FIP1L1::PDGFRA*), a favorable condition is presented in the sensitivity to TKIs; therefore, the first-line treatment is imatinib, with an initial dose of 100 mg/day. In case of resistance, the recommended dose is 0.30–0.40 g/day administered permanently. Imatinib offers 96% response rates and 88% progression-free survival from 3 to 5 years.

Hydroxyurea (hydroxycarbamide) is a commonly used antimetabolite in chronic myeloproliferative syndromes. It has a good cytoreducing effect, and the recommended dose is 20–40 mg/kg/day; nevertheless, the response rate may be lower. It is essential to mention that CEL tends to evolve into acute myeloblastic leukemia, and the prognosis is usually poor [16–18].

The case presented showed severe pulmonary involvement, necessitating assisted ventilation. Thus, intravenous cytarabine was used, and the response to the treatment was good, with a decrease in hyperleukocytosis and an improvement in lung function that allowed the discontinuation of ventilatory support. After the critical phase, treatment with hydroxyurea was continued, and a good hematological response was achieved for 24 months. The treatment of CEL is developing slowly because it is a rare disease. Recent reports have investigated the use of molecules, such as ruxolitinib—a JAK 1/2 inhibitor—for the treatment of CEL [19, 20]. Further extensive research is needed to evaluate these and other potential therapeutic molecules. This case report is relevant because it corresponds to a disease with complex characteristics, comorbidity, and reasonable response to cytarabine; however, it is essential to mention some limitations for future reports: (a) include a broader panel of possible fusions in CEL to improve diagnosis and therefore treatment, mainly due to the lack of recurrent and specific molecular events, such as *ETV6::ACSL6*, *ETV6::RAPGEF6* [21], *PDGFRA*, *PDGFRB*, or *FGFR1*, or with *PCM1-JAK2* [22, 23]; (b) additional cytogenetic and molecular genetic details are needed, but are lacking due to a lack of resources; (c) use clear thresholds for blasts, eosinophils, and organ damage considered by the International Consensus Classification of Myeloid Neoplasms and Acute Leukemia; and (d) limited patient follow-up.

“This case report was prepared following the CARE Guidelines.”

## 4. Conclusion

This case highlights the possibility of suspecting CEL through clinical manifestations, blood cytometry, peripheral blood, and BM morphology, allowing its diagnosis. Molecular cytogenetic studies allow for confirming the diagnosis, conducting differential diagnoses, and informing therapeutic decisions. All these actions are carried out to improve the patient's prognosis.

## Figures and Tables

**Table 1:** Blood test results.

**Test**	**Result**
Hemoglobin	8.5 g/dL
Absolute neutrophils	31.79 × 10^9^/L
Absolute eosinophils	69.40 × 10^9^/L
Leukocytes	105.97 × 10^9^/L
Lymphocytes	4.23 × 10^9^/L
Platelets	92 × 10^9^/L
LDH	940 U/L
PCR for *Mycobacterium tuberculosis*	Negative
HIV	Negative
HV B	Negative
HV C	Negative
PBS	Myelocytes, metamyelocytes, neutrophilia, and eosinophilia

Abbreviations: HIV, human immunodeficiency virus; HV, hepatitis virus; LDH, lactate dehydrogenase; PBS, peripheral blood smear; PCR, polymerase chain reaction.

## Data Availability

The data that support the findings of this study are available on request from the corresponding authors. The data are not publicly available due to privacy or ethical restrictions.
